# Bronchial intraepithelial recurrence of a pulmonary large cell neuroendocrine carcinoma

**DOI:** 10.1016/j.ijscr.2020.01.058

**Published:** 2020-02-06

**Authors:** Hideaki Kojima, Mitsuhiro Isaka, Kazuhito Funai, Yasuhisa Ohde

**Affiliations:** a Division of Thoracic Surgery, Shizuoka Cancer Center, Shizuoka, Japan; b First Department of Surgery, Hamamatsu University School of Medicine, Shizuoka, Japan

**Keywords:** LCNEC, large cell neuroendocrine carcinoma, HGNEC, high-grade neuroendocrine carcinoma, SCLC, small cell lung carcinoma, Case report, Large cell neuroendocrine carcinoma, High-grade neuroendocrine carcinoma, Recurrence, Bronchial intraepithelial spread, Bronchial intraepithelial recurrence

## Abstract

**Introduction:**

We encountered a case of a large cell neuroendocrine carcinoma with bronchial intraepithelial spread, a unique form of recurrence.

**Presentation of case:**

A 63-year-old man underwent middle lobectomy and systematic lymph node dissection for lung cancer. The pathological diagnosis was a large cell neuroendocrine carcinoma, and the pathological stage of the tumor was T2aN0M0, stage IB. At 30 months after surgery, the patient complained of continuous cough, and computed tomography revealed a thickened right upper bronchus. Transbronchial biopsy revealed a large cell neuroendocrine carcinoma with bronchial intraepithelial recurrence. The patient died of disease 24 months after recurrence was diagnosed.

**Discussion:**

In cases of centrally located small cell lung carcinoma, it is well known that tumor cells progress along the sub-bronchial mucosa. In contrast, the mechanism of tumor progression of large cell neuroendocrine carcinoma is not fully elucidated because of the rarity of such cases. Endobronchial metastases usually show the presence of polypoid lesions. To the best of our knowledge, this is the first case of endobronchial metastasis from a pulmonary large cell neuroendocrine carcinoma showing bronchial intraepithelial recurrence.

**Conclusions:**

Clinicians should be aware of this unique form of recurrence in patients with large cell neuroendocrine carcinoma of the lungs.

## Highlights

• We describe a pulmonary LCNEC with bronchial intraepithelial recurrence.
• Because of its rarity, the mechanism of tumor progression of LCNEC is still unknown.
• Endobronchial metastases usually showed the presence of polypoid lesions.
• This is the first case of metastasis with bronchial intraepithelial recurrence.
• Clinicians should be aware of this unique recurrence type in pulmonary LCNEC.

## 1 Introduction

Large cell neuroendocrine carcinoma (LCNEC) of the lungs was first proposed by Travis et al. in 1991 [1], and classified as high-grade neuroendocrine carcinoma (HGNEC) according to the World Health Organization International Histological Classification of Tumours [2]. Among cases treated with surgery, LCNEC accounts for approximately 3% of cases [3]. Because of its rarity, the clinicopathological features of LCNEC are unclear. In particular, the mechanism of tumor progression of LCNEC is still unknown. Herein, we describe a case of LCNEC with bronchial intraepithelial spread, a unique form of recurrence, and this work has been reported in line with the SCARE criteria [4].

## 2 Presentation of case

**Fig. 1:**
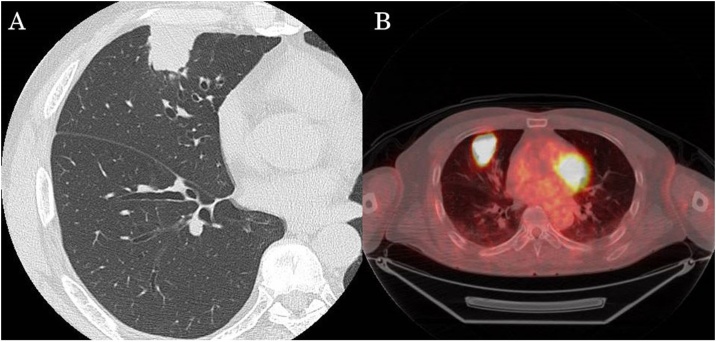
Radiographic findings of the tumor. *A*, Chest computed tomography revealed a 3.6-cm mass in the right peripheral S^5^ segment. *B*, On positron emission tomography, the mass showed ^18^F-fluorodeoxyglucose accumulation with a maximum standardized uptake value of 14.0.

**Fig. 2:**
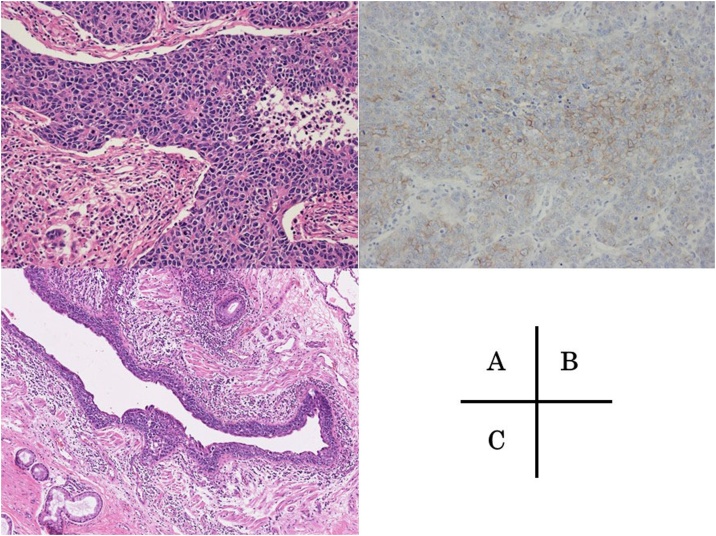
Pathological findings of the tumor. *A*, The tumor consisted of large cells with large round nuclei, distinct nucleoli, and scant cytoplasm, with many rosette-like structures. *B*, The tumor was diffusely positive for CD56. *C*, The bronchial epithelium around the primary tumor was extensively invaded by the tumor cells.

**Fig. 3:**
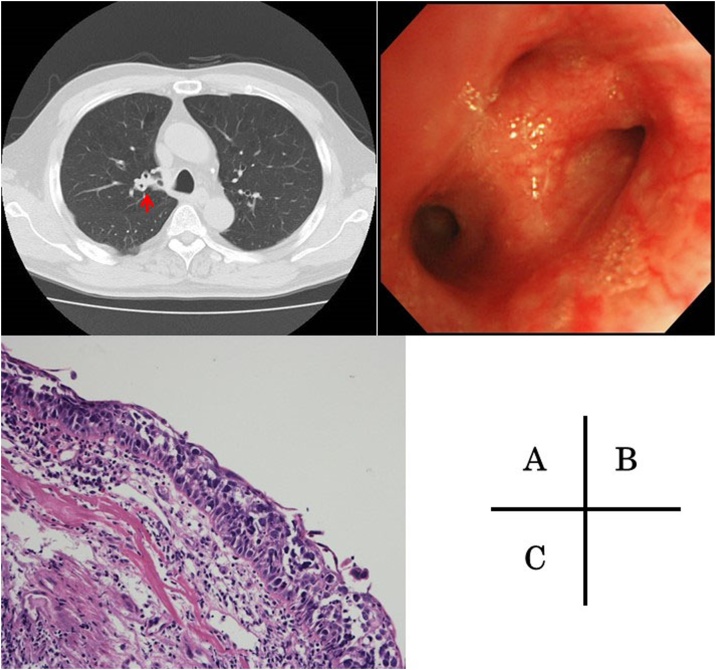
Clinical and pathological findings of the tumor recurrence. *A*, Computed tomography revealed a thickened right upper bronchus. *B, C*, Bronchoscopy revealed that the epithelium of the right upper bronchus was reddish and thick. Transbronchial biopsy yielded a pathological diagnosis of a large cell neuroendocrine carcinoma with bronchial intraepithelial recurrence.

A 63-year-old man was referred to our hospital with an abnormal shadow on a screening chest radiograph. He had smoked 2 packs of cigarettes daily for 40 years. Routine laboratory findings and the serum levels of tumor markers were within normal limits. Chest computed tomography revealed a 3.6-cm, well defined, lobulated, solid mass in the right peripheral S^5^ segment (Fig. 1A). On positron emission tomography, the mass showed ^18^F-fluorodeoxyglucose accumulation with a maximum standardized uptake value of 14.0 (Fig. 1B). The mass was identified as a non-small cell lung carcinoma on transbronchial lung biopsy and diagnosed as cT2aN0M0, stage IB. Accordingly, we performed right middle lobectomy and systematic lymph node dissection. The resected tumor was 4.0 × 2.5 × 2.0 cm with distinct margins. On microscopic examination, the tumor consisted of large cells with large round nuclei, distinct nucleoli, and scant cytoplasm, with many rosette-like structures (Fig. 2A). Immunohistochemical analyses showed that the tumor was diffusely positive for CD56 (Fig. 2B), and negative for chromogranin and synaptophysin. The final pathological diagnosis was LCNEC, and the pathological stage of the tumor was T2aN0M0, stage IB. In addition, the bronchial epithelium around the primary tumor was extensively invaded by the tumor cells (Fig. 2C). At 30 months after surgery, the patient complained of continuous cough, and computed tomography revealed a thickened right upper bronchus (Fig. 3A). Bronchoscopy showed that the epithelium of the right upper bronchus was reddish and thick (Fig. 3B). Auto-fluorescence imaging bronchoscopy revealed prolongation of the longitudinal folds. Transbronchial biopsy yielded a pathological diagnosis of LCNEC with bronchial intraepithelial recurrence (Fig. 3C), and the stump of the middle lobe bronchus was intact. Although the patient received radiation therapy, multiple liver and brain metastases developed and the patient died of disease 24 months after being diagnosed with the recurrence.

## 3 Discussion

LCNEC and small cell lung carcinoma (SCLC) are classified as HGNEC of the lung according to the World Health Organization International Histological Classification of Tumours [2]. In centrally located SCLC, it is well known that tumor cells progress along the sub-bronchial mucosa. In contrast, the mechanism of tumor progression of LCNEC is not fully elucidated because of its rarity.

We previously reported that the mechanism of tumor progression of peripherally located HGNEC might be bronchial intraepithelial spread but not to the sub-bronchial mucosa and hypothesized that LCNEC cells might have biological characteristics of bronchial epithelial cells [5]. In the current case, the bronchial epithelium around the primary tumor was extensively invaded by the tumor cells, which we referred to as “bronchial intraepithelial spread”. We made a diagnosis of LCNEC with bronchial intraepithelial recurrence because the stump of the middle lobe bronchus was intact and this lesion was a skip lesion only in the upper lobe bronchus.

Kiryu et al. reported that endotracheal metastasis is classified into the following 4 types: type I, direct metastasis; type II, bronchial invasion by a parenchymal lesion; type III, bronchial invasion by mediastinal or hilar lymph node metastasis; and type IV, peripheral lesions extending along the proximal bronchus [6]. Endobronchial metastases, in which the bronchial epithelium is directly involved, from primary lung cancer is extremely rare [6,7]. Furthermore, endobronchial metastases usually showed the presence of polypoid lesions. In the current case, transbronchial biopsy revealed LCNEC recurrence as bronchial intraepithelial spread not a polypoid or sub-bronchial mucosal lesion. To our knowledge, this is the first case of endobronchial metastasis from pulmonary LCNEC showing bronchial intraepithelial recurrence.

## 4 Conclusion

Clinicians should recognize this unique way of tumor progression as bronchial intraepithelial recurrence from pulmonary LCNEC.

## Sources of funding

This study did not receive any grants from funding.

## Ethical approval

This study was approved by the Shizuoka Cancer Center Institutional Review Board (29-J18-30-1-3).

## Consent

Written informed consent was obtained from the patient’ family for publication of this case presentation and accompanying images.

## Author contribution

HK designed the study, conducted the investigation, and wrote the manuscript. MI, KF, and YO supervised the work.

## Registration of research studies

This case report is not research study, therefore approval was not given.

## Guarantor

Hideaki Kojima accepts full responsibility for the work.

## Provenance and peer review

Not commissioned, externally peer-reviewed.

## Declaration of Competing Interest

The authors declare that they have no competing interests.
